# Culturally adapting relational savoring: A therapeutic approach to improve relationship quality

**DOI:** 10.1111/famp.12989

**Published:** 2024-03-27

**Authors:** Jessica L. Borelli, Elayne Zhou, Lyric N. Russo, Frances H. Li, Marta Tironi, Ken S. Yamashita, Patricia A. Smiley, Belinda Campos

**Affiliations:** 1 Department of Psychological Science, University of California, Irvine, California, USA; 2 Department of Psychology, University of Southern California, California, Los Angeles, USA; 3 Department of Social Ecology, University of California, Irvine, California, USA; 4 Department of Educational Sciences, University of Genoa, Genoa, Italy; 5 Department of Psychological Science, Pomona College, Pomona, California, USA

**Keywords:** attachment, cultural congruence, positive psychology, relational savoring, savoring

## Abstract

Relational savoring (RS) is a brief, strengths-based approach to heightening attentional focus to moments of positive connectedness within relationships. RS can be administered preventatively or within an intervention context when a therapist aspires to foster more optimal relational functioning. Typically administered within a one-on-one therapy setting, RS has demonstrated efficacy in enhancing intra- and interpersonal outcomes. To increase access to mental health services, the developers of RS are committed to engaging in an iterative approach of enhancing the cultural congruence and accessibility of this intervention within various cultural contexts, beginning with Latine groups in Southern California. In this article, we describe relational savoring and its theoretical and empirical support, including the process of culturally adapting the intervention within the context of three major studies, each with a distinct focus on Latine groups, a community that is underserved in mental health care settings. We then provide a vision for future research to improve upon the intervention’s compatibility for Latine families and other populations.

## INTRODUCTION

Developing interventions that are congruent with the values of communities that have been marginalized or have had limited access to mental health care is an important but sometimes overlooked aspect of intervention development (Park et al., 2023). The goal of achieving cultural congruence, for which there is no standard approach, may best be conceptualized within a flexible framework that guides the entire process of developing, testing, and delivering therapies, one that centers the perspectives and preferences of marginalized communities at key decision points. Here, we share the story of one intervention program – a brief, relationship-based intervention rooted in principles of attachment theory and positive psychology called *relational savoring* (RS) – in its ongoing quest to become more culturally congruent.

## SAVORING AS AN INTERVENTION APPROACH

Deeply immersing oneself in one’s positive experiences can provide immense psychological benefits. Bryant and Veroff (2007) refer to the tendency to attend to, appreciate, and even enhance one’s positive experiences as *savoring*. People who naturally engage in savoring report greater well-being and are less likely to have mental health problems (Hurley & Kwon, 2013; Smith & Hollinger-Smith, 2015). Although savoring is a trait-like emotion regulation tendency, it can also be taught as an intervention, yielding lower depression and greater happiness (Bryant et al., 2005; Hurley & Kwon, 2013; McMakin et al., 2011).

Bryant’s original conceptualization of savoring is general in its focus (i.e., people savor many different aspects of their experiences). However, given the centrality and importance of attachment relationships in people’s lives, as well as the potential for relationships to impact health and well-being (Mertika et al., 2020; Slatcher & Selcuk, 2017), attachment relationships may be a particularly important target of savoring interventions. Indeed, Fredrickson (2013) conceptualized love as consisting of micro-moments of positivity that resonate in every relationship. Noticing and preserving brief instances of connection and positivity through savoring helps make them less fleeting. Thus, RS interventions were developed to help people savor specific attachment-based experiences occurring within relationships (Borelli, 2024; Borelli, Smiley, et al., 2020).

RS is a brief, strengths-based program that can be used for prevention or intervention. It can be used as a stand-alone program or integrated into existing intervention programs to augment clients’ focus on positive connectedness. RS can be used as a prevention tool for populations who might benefit from enhancing their closeness or positive emotion within a targeted relationship (e.g., new parents, who may be at risk for relationship difficulties within the co-parenting relationship, the partner relationship, or the parent–child relationship). In research contexts, RS has typically been delivered in an individual therapy format by trained paraprofessionals (e.g., college-level students in psychology without clinical experience, lay community health workers),^1^ although it has also been delivered as part of group therapy (Borelli, Yates, et al., 2020), over the internet (Borelli, Rasmussen, et al., 2014; Burkhart et al., 2015), over the phone (Borelli, Sbarra, et al., 2014), and via telehealth (Nguyen et al., n.d.). RS can also be delivered by therapists in clinical practice as a stand-alone intervention or integrated into other intervention modalities, including individual therapy (e.g., as a complement to cognitive behavior therapy or emotion-focused therapy) and couples therapy (e.g., as a complement to Emotionally Focused Therapy, Greenberg & Johnson, 1988; for a review, see Borelli, 2024). RS may be particularly compatible with certain therapeutic modalities, including Acceptance and Commitment Therapy, which often incorporates mindfulness and savoring techniques as part of its values-based approach (Hayes et al., 1999), and with Emotion-Focused Therapy (Greenberg, 2004), which shares a strong focus on the importance of emotional expression. A therapist may wish to use RS as an intervention approach when either the client or the therapist identifies that the client would benefit from growth in the areas that RS targets – attachment security, closeness to others, reflective functioning, relationship satisfaction, and the expression of vulnerable emotions in the context of interpersonal relationship experiences. For instance, a therapist may wish to use RS when clients feel disconnected within a relationship, when there is a high level of conflict within a relationship, when a client has experienced a relational loss (e.g., death) or separation (e.g., work-related), or when a client feels rejected or unappreciated in a relationship. The RS therapeutic approach involves helping clients tune into their moments of positive connectedness with others, particularly during moments when they provided or received sensitive care to/from another person.

## RELATIONAL SAVORING PROTOCOL

When delivered in person in a one-on-one format, RS follows three steps. RS commences with a brief mindfulness exercise (step one) to reduce anxiety and stress, preparing the client for deeper reflection. In recent intervention trials (Borelli et al., 2019; Borelli, Hong, et al., 2022; Borelli, Kerr, et al., 2022; Borelli, Russo, et al., 2022), this exercise has taken the form of 1 min of paced breathing that the client and the intervener do together. Then, the intervener leads the client through memory selection (step two) and memory reflection (step three). In the memory selection phase (step two), the intervener helps the client recall two to three recent positive memories occurring with their relationship partner.^2^ The purpose of this phase is to identify the most optimal memory to savor. Once the client has shared a specific memory, the intervener asks questions to better understand the degree of specificity, positive emotionality, and emotional connectedness of the memory. The intervener also assesses the degree to which the memory contains difficult content or negative emotions that might intrude on savoring (which we refer to as “spoiling” in the manual;^3^ see Borelli, 2024). The treatment manual contains detailed instructions for how interveners can work with clients productively in the presence of negative emotion (see Borelli, 2024, for more details),^4^ but in brief, the approach involves validating the client’s negative emotion, creating space for the client’s negative emotion, and encouraging the client to explore positive emotions during the savoring session. Finding ways to simultaneously consider problems and difficulties while exploring positive emotional experiences and focusing on resources is a strategy shared by many intervention approaches (e.g., Boszormenyi-Nagy & Krasner, 1986). In the RS approach, if the intervener perceives strong negative emotions when the client recounts the memory, the intervener will help the client select a different memory. After memory selection (step two), the intervener and client move on to memory reflection (step three).

The memory reflection phase (step three) is a 5-step process in which the client sequentially focuses on different aspects of the memory. The five steps include: (1) Sensory reflection (i.e., client describes sensory details of the event), (2) Emotion reflection (i.e., client describes and re-experiences the emotions associated with the event), (3) Meaning making (i.e., client describes the thoughts associated with the event), (4) Future focus (i.e., client describes what the memory means about the relationship with their partner in the future), and (5) Mind wandering (i.e., client shares anything that comes to mind related to the memory). At each step of the process, the intervener draws the client’s attention to the precise moment when the client felt most connected to the other person. For instance, when reflecting on a loved one’s departure, the intervener might draw attention to the moment when the client’s partner pulled her back to give her one last embrace.

## THEORETICAL PREMISES OF RELATIONAL SAVORING

RS is a strengths-based program; throughout the interaction, interveners praise clients for the positive relationship behavior they exhibit, and at the same time, intentionally avoid commenting on undesirable behaviors. In this way, interveners selectively and positively reinforce the behaviors and emotions they want clients to re-experience; such comments also create a warm and supportive environment in which clients can explore their relational and emotional experiences.

RS is guided by two overarching theories, attachment theory and the broaden-and-build theory from positive psychology. Attachment theory (Bowlby, 1973) holds that experiences in early relationships lay the foundation for socioemotional wellness throughout the lifespan. Through early experiences with attachment figures, people internalize important messages about the self, relationships, and the world. If a caregiver provides a child with consistently sensitive care, the child develops secure attachment, learning that their needs will be responded to and that they deserve to be cared for. On the contrary, if a caregiver is inconsistently responsive to a child’s needs or becomes overwhelmed by their own emotions when responding to the child’s needs, the child becomes insecurely attached, perceiving that their needs are unimportant or dangerous and that the world is a frightening place where people cannot be counted on. Early relationship experiences set in motion a cascading series of internal processes – children build up a mental model of the self and the world that then causes them to interpret future relationship experiences in particular ways. Secure individuals are more likely to perceive experiences of loving care and concern as highly relevant and consistent with their worldview. For insecure people, these same experiences may be discounted as being irrelevant or the exception to the rule. Although internal working models (IWMs) of attachment (Bowlby, 1969, 1973) are thought to be adaptive in the context in which they develop, helping the individual acclimate to their particular relational environment, some IWMs may prevent people from getting possible benefits from positive aspects of their relationships. As with other interventions (e.g., security priming, Gillath et al., 2008), RS seeks to help correct this bias.

RS is premised on the belief that almost all individuals have moments of felt security in their daily lives, fleeting though they may be. For example, a person may catch their partner’s eye, even from across a room, and with one look, feel understood. Often, these moments pass by unnoticed, but moments of felt security – times when one feels safe, accepted, or deeply appreciated – are important to revisit for the positive emotion and feelings of connectedness they contain. Although many people have a threat bias (Mulckhuyse, 2018), those with attachment insecurity may be even more strongly predisposed to lean this way and thus may stand to benefit the most from the RS intervention. Notably, the experience and attribution of threat are affected by multiple internal and external factors. For example, minoritized populations may be more likely to experience collective threats that target their social groups (Cohen & Garcia, 2005). When interventions are developed with cultural congruence in mind, attention to specific threats and specific sources of strength can be incorporated. Further, individuals residing in unsafe environments, such as those characterized by live threats (e.g., abuse, domestic violence) must be vigilant to threat in order to be safe. They may not be suitable for this approach, a limitation that will be discussed in depth below.

The second theoretical framework underlying RS is the broaden-and-build theory (Fredrickson, 1998) within positive psychology (Seligman et al., 2005). This perspective holds that positive emotional states have a beneficial impact on psychological functioning. Specifically, being in a positive emotional state opens people up to considering new perspectives, approaching problems from new angles, and being more creative (Fredrickson, 2001). Positive emotions can also help people recover from negative emotions more quickly and effectively (Fredrickson, 2005). For instance, practicing gratitude (e.g., writing and then sending a letter of gratitude to a person who has been kind) has the power to increase happiness, decrease depressive symptoms, and produce positive changes (Seligman et al., 2005). In contrast, being in a negative emotional state can constrict these same psychological capacities, making people more inflexible or rigid (Fredrickson, 2004).

These principles are similar to those that underlie mindfulness-based programs; that is, being in a euthymic state can help relax one’s cognitive processing and open one up to a more reflective space (Kabat-Zinn, 2003). These ideas have been supported by recent neuroscientific developments (e.g., Hanson, 2016) showing that, to preserve the species, the human brain is oriented toward negative psychological experiences (e.g., threats) to keep physical danger at bay. Noticing and savoring positive experiences and emotions does not appear to be a biologically inscribed behavior in human beings, but rather a learned ability that nevertheless has enormous benefits (Hanson et al., 2021). Based on these findings, RS begins with a brief mindfulness exercise to reduce physiological reactivity and promote expansive reflection before proceeding with the intervention. Further, by focusing on positive relational experiences (as opposed to negative ones), we seek to enhance openness to psychological growth (new emotions, new perspectives on the self and other).

### Connection with existing family and couple therapy theoretical frameworks

Although the roots of RS can most closely be traced to attachment and the broaden-and-build theory of psychology, its tenets also have connections with other theoretical traditions within couple and family psychology. For instance, in line with a basic principle of attachment theory (Bowlby, 1969, 1988), RS positions the provision and receipt of care as reciprocal influences, whereby increases in security in one dimension (e.g., caregiving) will influence the other (e.g., care-receiving). This stance is consistent with the moral theory of care ethics, which holds that the roles of care-receiver and caregiver are intertwined and influence one another (Collins, 2015). Further, originating from Gilligan’s work (Gilligan, 1993), the moral theory of care ethics emphasizes the primacy of relationships as a fundamental context for making ethical decisions, recognizing that individuals are interdependent and need to rely on and care for others in order to achieve well-being.

Focusing on interdependence as a therapeutic goal has parallels with other schools of thought within family and couple therapy. The construction of the relational self has been discussed by Fishbane (2001) and Gergen (1991). Fishbane (2001) argues that developing a relational self-narrative can promote interest in and empathy for the client’s perspective as well as that of the other, and help to overcome the central mentality within family conflicts of competing interests or power struggles. Relatedly, expanding client awareness by constructing stories narrated from different benevolent sources is the main goal of narrative therapy (White, 2007). Similarly, RS encourages clients to narrate their experience through the gaze of a person who has or has had a loving and benevolent investment in them. Approaches to couple and family therapy also seek to shift a client’s focus from negative tracking of a partner’s or child’s misbehavior to one in which they identify positive behaviors (Epstein et al., 1993). RS aligns with these approaches, with the aim of shifting the individual’s attention to positive perceptions and the positive responses they give and receive from significant others (Borelli, 2024).

Finally, RS is consistent with many of the central aims of Emotionally Focused Therapy (EFT; Greenberg & Johnson, 1988) which hold that the expression of vulnerable (primary) emotions, particularly those relevant to attachment needs, is important for healthy psychological functioning. Likewise, a central premise of RS is that expressing vulnerable positive emotions related to attachment relationships (e.g., feeling understood, safe, accepted; feeling loved, cherished, and secure) grounds the individual in their feelings of security. In EFT, the expression of emotions occurs in the context of couples therapy, while in RS, the expression of vulnerable emotions typically occurs between client and therapist (though RS has been conducted between couples in clinical but not research contexts; see Borelli, 2024, for an overview). Both approaches hold that sharing these vulnerable states will strengthen attachment bonds, but notably, EFT focuses on the expression of unmet attachment needs while RS focuses on attachment needs that have been satisfied.

## EMPIRICAL SUPPORT FOR RELATIONAL SAVORING

The efficacy of RS has been examined in several studies with a variety of populations. The findings reveal that RS is effective in improving emotional states (Borelli, Kerr, et al., 2022; Burkhart et al., 2015), relationship satisfaction (Borelli, Rasmussen, et al., 2014), parenting sensitivity (Borelli, Hong, et al., 2022; Borelli, Kerr, et al., 2022; Borelli, Russo, et al., 2022), and cardiovascular reactivity (Borelli et al., 2019), and when delivered in the context of an attachment-based group intervention, in reducing mental health symptoms (Borelli, Yates, et al., 2020). RS has been evaluated among parents (majority female) of toddlers (Borelli et al., 2023; Borelli, Kerr, et al., 2022; Burkhart et al., 2015; Doan et al., 2023; Smiley et al., 2024), incarcerated parents (Kerr et al., 2022), adult romantic partners in long-distance relationships (Borelli, Rasmussen, et al., 2014), partnered couples (Wang et al., 2023), female non-deploying partners of military personnel (Borelli, Sbarra, et al., 2014; Froidevaux et al., 2023), older adults aged 60 to 90 (Borelli et al., 2019), adolescent males in residential treatment (Wang, Bouche, et al., 2020; Wang, Huang, et al., 2020), and low-income Latine^5^ mothers and adolescents (Borelli, Yates, et al., 2020). The intervention has also been evaluated among parents of children with autism spectrum disorder (Gaskin, 2021; Pereira et al., 2021) in the United States and Singapore. In addition, several of these studies have examined moderators of response to the intervention or predictors of engagement with the intervention. On the one hand, clients with greater risk (e.g., higher attachment avoidance, higher depressive symptoms) show greater response (Borelli, Hong, et al., 2022; Burkhart et al., 2015), perhaps because their RS is lower quality to begin with (Bond & Borelli, 2017; Borelli, Hong, et al., 2022). On the other hand, adults who begin an intervention trial with higher relationship satisfaction or lower attachment avoidance (Borelli, Rasmussen, et al., 2014; Borelli, Sbarra, et al., 2014) also benefit more from RS. In sum, these preliminary findings suggest the short- and medium-term efficacy of this intervention/prevention approach.

In constructing RS, several goals were at the forefront. First, the developers sought to construct an intervention that was not only grounded in psychological principles but also scalable. That is, designing a protocol that could be easily delivered by interveners without advanced training (e.g., lay community health workers, paraprofessionals) would enhance its potential for dissemination. Second, it was important that the intervention be acceptable to underserved populations. Early findings suggested that it might be particularly appealing to a specific subpopulation in southern California. Goldstein et al. (2019) conducted a re-analysis of data from Burkhart et al. (2015) and found that Latine parents high in attachment avoidance increased parental reflective functioning (a measure of mentalizing) following RS, whereas non-Latine parents did not. We reasoned that for high avoidance Latine parents, the fact that the intervention was culturally matched with values that are strongly held within many Latine families might have contributed to the intervention’s impact. For instance, RS is consistent with values of *familismo*, the prioritization of family relationships (Corona et al., 2017), and *simpatía*, an emphasis on harmonious social interactions characterized by positive emotional expression (Acevedo et al., 2020). The focus of RS on close relationships and positive emotions may naturally resonate with these values, perhaps making RS more relevant to Latine parents. In other words, even though the intervention approach may have been at odds with their avoidant attachment style, the fact that it fit with their cultural values may have made it more likely for them to benefit from it. Although not initially developed with Latine families in mind, this exciting finding encouraged us to examine the efficacy of RS for Latina mothers as an a priori goal in a subsequent trial of RS and to begin developing a culturally congruent intervention/prevention program in earnest. When embarking upon this goal, we felt that it was important to note that Latine individuals vary in terms of the extent to which they would benefit from cultural modifications of an intervention; some Latine individuals who are highly U.S. acculturated may not need or benefit significantly from a highly adapted intervention, whereas Latine individuals who are less U.S. acculturated may benefit from an intervention approach that has been more extensively adapted. In our work, we have partnered with communities that vary in the extent to which they are likely to benefit from adaptation of the intervention. Below we present a guiding framework for working toward cultural congruence, illustrated via several case studies.

## STEPS ON THE PATH TOWARD MAKING RS CULTURALLY CONGRUENT

Culturally congruent care, as defined by the American Nurses Association standards of practice, is “in agreement with the preferred values, beliefs, worldview, and practices of the healthcare consumer” (Marion et al., 2016). For a client to receive culturally congruent therapy, the therapy itself must be both acceptable and accessible. In the following section, we outline lessons learned and active steps taken to enhance the cultural congruence of the RS intervention for Latine families. Although we refer to a broad “Latine community” in our discussion, we also acknowledge the remarkable heterogeneity within groups that may be labeled with this term, and the importance of tailoring interventions to individuals or groups as needed. We have conducted RS with a diverse composition of samples, but we focus on Latine families as this is the underrepresented minoritized group with whom we have had the most in-depth experience. Further, it is the largest racial-ethnically minoritized group in the geographic region where we work.

In the sections that follow, we present three studies that illustrate the path we have taken in adapting RS to be culturally congruent for Latine families. In making modifications to the intervention, we have focused on two types of adjustments. First, we have attempted to adapt RS so that it more fully embraces the values of the community being served (i.e., broader cultural values, inclusion of local metaphors). We refer to this as a cultural adaptation. This type of adaptation is more likely to be specific to the cultural group targeted (in this case, to members of the Latine community). Second, we have worked to increase the accessibility of the intervention, which removes barriers to dissemination – this type of adaptation can be advantageous for many different cultural groups (i.e., not just members of the Latine community). We refer to this as an accessibility adaptation. Below we present a brief description of each study and its findings, followed by the concrete steps we took to culturally adapt RS, as well as the outcomes of the adaptation process. Table 1 presents a summary of the modifications for each study.

### At-home intervention for mothers of toddlers: The PARENT study

#### Brief overview

Our journey begins with the examination of a version of RS that had not been culturally adapted at all – in fact, the first illustration of RS involves the lessons we learned from administering the de novo intervention to a sample that included Latine participants. The Promoting Attachment and Relational Engagement with Toddlers (PARENT) Study (IRB *#4/29/2016JB-MP*; Borelli, Kerr, et al., 2022) was a randomized controlled trial that examined the immediate and long-term impacts of RS among mothers of toddlers (aged 18–27 months) with a high percentage of Latina (41%) participants due to the demographic composition of the area where the families resided. In this study, the RS protocol and the savoring exercises were focused on the mother–child relationship. The directions prompted mothers to focus on a time when they felt close, connected, or in sync with their child; a time when they supported their child, or a time when their child needed comfort and they were there to provide it.

##### Procedures

Following a baseline assessment, mothers were randomly assigned to receive four 30- to 45-min sessions of either RS or personal savoring (a control condition in which mothers were asked to savor a positive experience they had by themselves; e.g., listening to a good song, getting a promotion). Conversations with mothers prior to launching this study suggested that delivering interventions in the home and during the early evening would increase accessibility. As a result, home visitors delivered interventions in mothers’ homes during the evening hours. We assessed outcomes both immediately following the intervention phase of the study and at three-month follow-up.

##### Results

All mothers who received RS reported higher positive emotion (e.g., gratitude and pride) and closeness to their child immediately following the savoring sessions, and displayed greater parenting sensitivity at follow-up. Latina mothers, in particular, showed greater reflective functioning and greater use of the intervention (i.e., intervention uptake) at follow-up (Borelli, Kerr, et al., 2022). Examination of potential mechanisms underlying treatment effects is underway (Borelli et al., 2023; Borelli et al., 2024).

#### Practices in service of cultural congruence

##### Cultural adaptations

Though cultural congruence had not been a primary stated goal and we did not make any adaptations to the intervention to increase its alignment with cultural values, the findings of the PARENT study revealed that the intervention may have been particularly acceptable to Latine families. Acceptability refers to the degree to which a therapy is compatible with the population of interest and meets its needs (Ayala & Elder, 2011). We were therefore curious to uncover aspects of the intervention that may have particularly resonated with this group. One hypothesis pertained to the alignment between RS focus and cultural values long linked with Latine groups such as *familismo* and *simpatía*. The resonance of RS with this group’s values was apparent in the content of the savoring narratives themselves. That is, Latina mothers’ narratives demonstrated themes of familial connectedness and positivity within relationships (Borelli, 2024). Further, Latina mothers showed higher levels of reflective functioning – curiosity about their children’s internal experiences – at the 3-month follow-up (Borelli, Kerr, et al., 2022).

##### Accessibility adaptations

Efforts made in the PARENT Study toward accessibility were likely important in enhancing the cultural congruence of the intervention. That is, the intervention was intentionally designed to be administered by non-expert interveners (e.g., college students) after a brief training process, with the goal of reducing barriers to access such as requiring a certain educational level or intensive training that could impede therapy delivery. Upon examining the fidelity of the intervention, we found that paraprofessionals with limited prior experience and training delivered the RS intervention with high fidelity. Indeed, interveners without bachelor’s degrees delivered sessions with higher fidelity than those with bachelor’s degrees, further enhancing confidence in the potential of this intervention for implementation and dissemination (Borelli et al., 2024).

In addition, the PARENT Study made the RS intervention accessible through brief sessions conducted in mothers’ homes, usually in the evening hours while their children were asleep. This was based on feedback provided by parents, as well as the rationale that for parents of young children, this time presents a rare opportunity to reflect inward. Further, providing intervention sessions at home reduces the burdens of childcare and transportation. It is worth mentioning that these aspects of the intervention increase its potential for dissemination for many different populations, not just Latine families. However, particularly when it comes to working with under-resourced communities, such as lower-income Latine groups, reducing barriers to accessing care increases cultural compatibility.

#### Case study

The following excerpts from the PARENT Study illustrate the process of the brief RS intervention for mothers of toddlers. Over 4 sessions, this mother, who identifies as Latina, demonstrated powerful growth by incorporating the reflection process into her daily life as a parent.

##### Session 1

###### Intervener:

Finally, is there anything else you would like to say about this memory or do you have any take-aways from engaging in this reflection process? *Client*: I never really thought about it, [laughs] until I thought, you know, when I started thinking about the future, how what I do now is important to the relationship I’m going to have with her. When she’s older … I never really reflected. And again don’t have time [laughs] I’m sure you hear that from a lot of parents. You just don’t have time when you’re in the middle of it. You just don’t have the time to think about it.

##### Session 4

###### Intervener:

Finally, is there anything you’d like to say about this memory, or do you have any takeaways from engaging in this reflection process? *Client*: I enjoy being able to reflect upon it. Because life happens so fast. And, you really don’t, sometimes, think about some of the things that you do and why you do them when you’re caught in the moment. And I enjoy that I have been able to just think about my interactions with her and how I approach her sometimes. You know, ‘cause sometimes it can be difficult [smiles]. But also, I like that I can think about the positive things that we get to do together.

In sum, from the PARENT study, we learned that our un-adapted intervention was already congruent in many ways with the important values of many Latine families. Further, the mode of delivery of the intervention (home visits, use of paraprofessionals, delivering visits in evening hours), designed to increase accessibility, may have boosted the appeal for Latine families in particular. That is, increasing accessibility might have increased trust in the interveners for a cultural group that experiences higher levels of mental health stigma that often impede help-seeking behaviors (DeFreitas et al., 2018; Zhou et al., 2021).

### Co-developing an RS-based group intervention: The Confía en mi, Confío en Ti study

#### Brief overview

Following the promising findings of the PARENT study, when we set out to develop a new intervention, we were determined to explicitly build cultural congruence into the intervention protocol, beginning with the process of intervention development. This study constituted the first attempt to proactively adapt RS. The process involved an 18-month intervention adaptation period, wherein we partnered with Latino Health Access (LHA), a local community agency primarily serving low-income Latine families, to co-develop, implement, and test the effectiveness of an 8-week group intervention for Latina mothers and their children (aged 8–17 years; UCI IRB HS# 2017–3974). Latino Health Access is an agency led by *promotores* (i.e., trained community health workers or promoters of community health), who by definition are part of the community they serve; they contribute to the development, design, and dissemination of research and service delivery programs (Bracho et al., 2016). We developed an academic-community partnership of researchers (White and Latine academic psychologists) and administrators, research assistants, and *promotores*. Prior to initiating this work, the researchers who did not belong to the target population reflected on approaches that would reduce the likelihood of researcher bias or unintentionally cause harm to communities. We intentionally adopted a community-based participatory research (CBPR) framework, as applied in another evidence-based parenting intervention targeting Latine families (Parra Cardona et al., 2012, 2023). CBPR aims to share power and collaborate in partnership with community members, who are the experts of their own lived experiences (Wallerstein & Duran, 2006; White & Epston, 1990).

LHA collaborators were included as part of the research team from the start; they provided integral feedback on what became the *Confía en Mí, Confío en Ti* intervention, which translates to “Trust in me, I trust in you.” The resultant intervention is a group-based therapy approach to address attachment, self-efficacy, and prosocial/anti-violence norms in Latine youth and their caregivers delivered over eight consecutive weekly sessions (Borelli, Yates, et al., 2020). Mothers and youth attend separate groups operating in parallel in which they learn about core attachment principles, such as the importance of close connection for feeling safe in the world, the emotional needs of adolescents, and how primary caregivers can support adolescents exposed to adversity. RS is a focal point of the intervention for both mother and youth groups, with 2 of the 8 sessions dedicated entirely to the practice. After learning about the importance of attachment relationships, mothers and their teens savor the safety/security they provided (mothers) and received (youth) from one another.

Promotores from LHA worked with our research team to revise and adapt the RS manual to best meet the needs of the community members the intervention aimed to serve. This extensive, necessary process integrated several CBPR practices to enhance the cultural congruence of our intervention, including prioritizing community members’ feedback and knowledge (Borelli, Cervantes, et al., 2021; Borelli, Russo, et al., 2022; Infante et al., 2011), tailoring the manual to align with cultural values and needs, and building trusting partnerships with community members (Messias et al., 2013). Importantly, promotores led the intervention, a decision made for two reasons. First, as trusted community and ingroup members, promotores were most appropriately equipped to elicit “buy-in” from participants and promote long-term adherence to treatment protocols. Second, involving promotores would facilitate the sustainability of the intervention once the research study concluded; it was essential that these trusted community members could take skills from the intervention with them to maximize its reach in the community. The use of promotores in treatment co-development and delivery phases was an important part of the intervention adaptation.

##### Procedures

Mother-youth dyads, recruited from the community by the promotores, were randomly assigned to either the intervention or a waitlist control group before completing a pre-treatment assessment. They then participated in the community intervention (8 weekly, 2-h group therapy sessions led by Latino Health Access promotores). The mothers’ group was delivered in Spanish while the youth group was delivered in English. Following the intervention, dyads completed ipost-treatment and 6-month follow-up assessments.

The resultant sample consisted of *N* = 330 mothers (*M*_age_ = 40.54, *SD*_age_ = 6.48) and youth aged 8 to 17 (*M*_age_ = 12.28, *SD*_age_ = 2.16; 51.4% male). On average, mothers had 3.13 children and had completed eighth grade (*SD* = 2.90 years). The majority of mothers (97.4%) were not born in the U.S. (90% from Mexico, 2.4% from El Salvador, 1.2% from Guatemala, and 0.3% from Columbia) and primarily spoke Spanish (97.4%). Youth (95.2% U.S.-born) primarily spoke both Spanish and English at home (73.6%). Mothers reported a median household income of $24,000/year.

##### Results

Within the intervention group (*N* = 112), youth aged 8 to 13 years showed increases in attachment security from pre-treatment to post-treatment, while adolescents aged 14 to 17 years showed increases in reflective functioning (Borelli, Yates, et al., 2020). In terms of mental health outcomes, youth symptoms (depression, anxiety) decreased from pre-treatment to post-treatment, as did maternal depressive and anxiety symptoms (Borelli, Yates, et al., 2020). These outcomes were assessed after the entire 8-week intervention, not just after two of eight sessions focusing on RS, so they should be interpreted with caution; that is, positive effects could be due in part to other aspects of the intervention, for example, group discussions of attachment principles or the work on social determinants of health. Further, due to difficulty retaining a waitlist control sample (owing to COVID and other retention difficulties), we were only able to examine pre-post intervention outcomes in the treated sample, further limiting the conclusions we can draw from these findings. However, our results suggest there are specific contributions from RS interventions within community-based programs.

#### Practices in service of cultural congruence

##### Cultural adaptations

The team took several steps to adapt the intervention in alignment with cultural values. This process involved a dynamic collaborative partnership with Latino Health Access that began prior to the intervention administration. First, we introduced a tentative idea for the protocol, and then LHA’s promotores highlighted what was likely to resonate with the community and what needed further refinement. In this crucial co-development stage, the research team worked with LHA collaborators to interlace cultural considerations with therapy content. We made adjustments to the RS protocol to ensure an authentic resonance with the community’s values and cultural expressions, including, for example, attending to the length of the instructions, how RS was introduced (via a script delivered orally along with a handout with images and a few written words), what resources would be provided to families, and how to go about the memory selection stage. With regard to memory selection, promotores shared their concerns that the prevalence of trauma exposure among the families they serve could impact a person’s ability to reflect on a safe haven/secure base interaction with their child/caregiver. In response to this insight, we discussed strategies that would allow participants who found it difficult to identify a positive memory from their past to comfortably participate in RS while maintaining sensitivity to their circumstances and not drawing undue attention to them. Together, we devised a refined protocol where individuals were presented with the option to savor an experience they wished they had with their own caregiver (but never actually had). In other words, they were given the option of savoring a “wished for” experience with a caregiver. This modification to RS ended up being extremely helpful; several participants began savoring “wished-for” memories and then transitioning into savoring real memories with caregivers. Importantly, in subsequent sessions, they were able to effectively savor positive memories with their own children without negative memories from their past intruding, which had been a concern during the intervention co-development phase.

In addition to the above changes to the protocol during the development phase, during implementation, we undertook several additional adjustments to ensure that the RS sessions were appropriate for group contexts and met the needs of Latine participants. For the mother groups, this entailed having the promotores who facilitated the groups introduce the main concepts and reasoning underpinning RS (namely, “*el disfrutar la relacion*” which translates to enjoying the relationship) before starting the savoring exercise. This introduction was designed to resonate with relevant cultural perspectives and values (e.g., familismo, simpatía) of Latina mothers – highlighting the value of positive emotions and relationships as a source of strength (Corona et al., 2017; Senft et al., 2021, 2023) – as well as to demystify the intervention procedures (Rhodes et al., 2018). Culturally relevant materials were created or borrowed to facilitate deeper and more meaningful engagement by mothers. For instance, we presented short videos in Spanish illustrating secure base and safe haven behaviors from the Circle of Security intervention (Marvin et al., 2002) program’s freely available resources. We also introduced an integrated tree metaphor (where the trunk, branches, leaves, and roots of a person’s tree represented the person’s strength’s goals, and support system). Promotores also presented a video-recorded RS intervention in Spanish as an example and tangible aid to the mothers. Finally, the promotores led the mothers in a short breathing exercise to help calm their bodies before asking them to complete the RS exercise in pairs. Pairs were provided with handouts (in Spanish) that briefly detailed the steps of RS, using language and examples that were culturally relevant. By providing these take-home resources, we aimed to facilitate mothers’ practice of savoring during the intervention at LHA and at home following the intervention. The iterative process continued throughout the project period, fostering a collaborative, shared vision that culminated in an RS manual that was a true reflection of our collective insights and understanding of the families we aimed to serve (Borelli, Cervantes, et al., 2021; Borelli, Russo, et al., 2022; Borelli, Yates, et al., 2020).

Similarly, youth were taught in one session about the secure base concept and asked to savor in pairs an experience when their mother acted as a secure base for them. In a subsequent session, they explored the safe haven concept and were guided in savoring an experience when their mother served as a safe haven. The use of handouts and worksheets was again a conscious choice for the youth groups; we anticipated that untrained peers participating in the intervention would require more structured guidance compared to adult interveners. These materials visually represented the most salient aspects of the intervention, making it highly accessible to youth. Further, the intervention language and protocol were simplified to ensure that participants focused on savoring their memories rather than feeling pressured to get everything right.

##### Accessibility adaptations

In addition to incorporating procedures and content to address cultural values, the involvement of promotores in the delivery of the intervention extended the acceptability and accessibility of RS (Messias et al., 2013), making the program responsive to community needs. Among the most common barriers to general healthcare access are language barriers and, particularly in the context of mental healthcare, stigma (Golberstein et al., 2008; Handtke et al., 2019). For many racial-ethnic minoritized clients, the healthcare system may be especially difficult to navigate when culturally sensitive healthcare providers (Handtke et al., 2019) and professionals from their own racial/ethnic groups (Salsberg et al., 2021) are scarce. Promotores, as valued members of the community they serve, offered cultural and linguistic alignment that resonated deeply with participants in the present study. Cultural congruence was further bolstered by their status as non-therapists, reducing fears of stigmatization that may come from participating in behavioral/mental health interventions (Barnett et al., 2018). Following the completion of the study, promotores (*N* = 8) completed interviews and assessments to share their thoughts and gauge the impact of the intervention. Their feedback revealed a sense of fulfillment and reward in delivering the program, affirming the value it brought to the community and to themselves (Borelli, Russo, et al., 2022). Moreover, promotores provided valuable recommendations for future iterations of the intervention to better address the unique needs of the youth involved (e.g., greater use of visual aids), as well as to facilitate and increase participant retention (e.g., shorter sessions, fewer sessions for youth).

#### Case study

In this study, we respected community members’ requests to not record participant sessions and instead relied on promotores’ reports and qualitative data to offer a nuanced case study showcasing the intervention’s effectiveness (Borelli, Cervantes, et al., 2021). From their feedback, it became evident that mothers, in particular, derived substantial benefits from the intervention (Borelli, Russo, et al., 2022). They acquired practical strategies that improved their relationships with their adolescent children, including a deeper understanding of their children’s developmental needs and a shift in how they communicated with their children. Significantly, the attachment-based content within the curriculum played a pivotal role in this transformation, helping mothers identify the importance of being a secure base and acting as a safe haven for their children during times of need. This is illustrated by the following quote (translated from Spanish) from a mother sharing how the intervention positively impacted her relationship with her child: “I felt really good because my son and I have had better communication and we continue to have communication and honestly I am very grateful to you all and this class that you gave us. I am honestly happy and now my son is always like ‘Let’s go to the group and at the beginning, he did not want to. He sees that the environment is different and it has helped him. It has also helped me as a mother and I also like that no one judges each other and we all help each other. I really enjoyed it.”

In sum, this collaboration yielded a comprehensive cultural adaptation of the intervention. The community partner team had equal status and voice in the collaboration, partnering with the research team from the inception of the study.

### Adapting RS for Latine Lay Health Workers: The El Corazón de la Comunidad Study

#### Brief overview

Our ongoing investigation of RS aims to extend the scope of the intervention beyond parents and youth to include promotores/community health workers (CHWs)^6^ themselves (UCI IRB #1596). Outcome results are not yet available because this study is still in progress. This shift was inspired by the positive feedback from LHA CHWs who shared that they had integrated the practice into their personal lives (Borelli, Russo, et al., 2022). Further, as part of the immense toll of COVID-19 on Latine people generally, one of the hardest hit groups was CHWs (Marquez et al., 2023). We witnessed firsthand the CHWs’ indispensable role in safeguarding their communities and it inspired us to create a space for them to savor moments of providing care, thereby affording them an opportunity to reflect on the positive impact they have on those around them (Arcos et al., 2023). Aptly named the *El Corazón de la Comunidad* (i.e., “Heart of the Community”) study, this adaption of RS instructs CHWs to focus on times when they provided secure base support (when they assisted someone in achieving a goal or meeting a milestone), safe haven support (when they provided emotional support to individuals navigating difficult situations) or moments of connection with community members. The structure (4 consecutive weekly sessions) is similar to that of the PARENT study, with interveners (bilingual/bicultural research assistants, mostly undergraduate and post-baccalaureate students) facilitating the RS sessions one-on-one. As one of the main aims of this ongoing study, we seek to test whether RS will benefit CHWs’ mental and physical well-being, ultimately increasing job satisfaction, mitigating burnout, and improving cardiometabolic health (Arcos et al., 2023).

##### Procedures

In this study, CHWs were randomly assigned to the intervention or waitlist control group before completing a pre-treatment assessment and then beginning the intervention. Following the intervention, CHWs completed a post-treatment assessment and a 3-month follow-up assessment (Arcos et al., 2023).

Although this study is ongoing, the current baseline sample consists of *N* = 75 Latine CHWs (*M*_age_ = 42.62, *SD*_age_ = 12.47, 92% female). On average, CHWs have completed twelfth grade (*SD* = 2.67), have been employed at their agency for 7.29 years (*SD* = 5.58), and have an annual household income of $44,249 (*SD* = $21,439). The majority of CHWs (87%) were not born in the U.S. (87.0% from Mexico, 8.7% from El Salvador, 2.2% from Venezuela, 2.2% from Peru), had children (94.7%), and reported being married (68.0%).

#### Cultural congruence practices

##### Cultural adaptations

The shift in our focus to CHWs once again presented us with the opportunity to enhance the cultural congruence of this iteration of RS, which we began by focusing on alignment with cultural values. The research team revised the protocol using feedback from interviews with LHA CHWs before the beginning of intervention administration (Infante et al., 2011). The research team’s modifications to the RS manual included adjustments to ensure that the themes of interest (secure base and safe haven support, moments of connection) were reflective of CHWs’ encounters with community members and that the examples used would resonate with their personal experiences to create a sense of authenticity within the intervention. Unlike traditional service providers, CHWs are pillars of support, advocates, and trusted individuals within their communities (Infante et al., 2011), so we customized RS to reflect their distinct roles and close relationships with community members. We modified the protocol to include terminology and phrasing that CHWs commonly use to describe their work. Including relevant examples of the principles we aimed to teach and incorporating familiar terminology improves the match between this approach and the community’s cultural values. Through this process we sought to enhance the relevance of the intervention to the unique roles of the promotores, thereby strengthening the likely impact on its capacity to bring about positive impacts on their mental and physical well-being. After making these modifications and prior to beginning the study, we reviewed this revised intervention protocol with a different group of promotores who were involved in leading the grant-funded effort with our team.

##### Accessibility adaptation

In addition to the modifications we made to the RS protocol to enhance its adherence to cultural values, we also made modifications to enhance its accessibility. In designing the study, our conversations repeatedly turned to the challenge of attrition. Clients often found it difficult to commit to the rigid schedules of in-person interventions, which could be burdensome given their life demands. In the broader context of inequitable access to healthcare, lack of transportation has also been implicated as a major barrier to receipt of care (Institute of Medicine [U.S.] Committee on Understanding and Eliminating Racial and Ethnic Disparities in Health Care, 2003). This, along with concerns around COVID-19 transmission, led us to conduct all sessions virtually to enhance accessibility and flexibility.

#### Case study

The following excerpts (translated from Spanish and anonymized) from *El Corazón de la Comunidad* illustrate the process of RS sessions for CHWs. Across sessions, this CHW, who identifies as Latina, illustrates growth in her savoring content.

##### Session 1

###### Intervener:

And now I’d like you to think about what you were thinking [at the youth outreach event]. *Client*: Definitely happy to be able to do something new with them, for them. Also, just thankful that I am able to have this role in the community, especially with these youth at this time in their lives. I remember what it was like to be a youth. So, I think it’s important. My role and my coworkers were really important. Also being a mentor for them at this time. And yeah, just thankful for the fact that I’m able to do what I do.

##### Session 3

###### Intervener:

Now I’d like you to think about what you were thinking when you were in your office meeting with your youth. *Client*: Yeah, I definitely feel happy. Happy to have the personality that I have that allows the youth to feel comfortable to come up to me. But I also just feel happy that the youth are continuing to go because they could easily say, “You know what, I don’t want to go to the meeting,” but they decide to go because they know that it is a safe space for them. And I’m happy that myself and my teammates provide that space and emotional support that they need. They’re going through a lot, definitely. And sometimes they don’t feel comfortable sharing it with their parents, or maybe they do share it with their parents. But their parents are not fully able to resonate with them, because their path has been different. So I love the fact that probably our team, the youth team, probably experienced similar challenges to the youth. So we’re able to make those connections more easily with them, especially with these situations about school internships, like all of those things. So I definitely feel like I play an important role in this community because I am able to support them.

## FUTURE DIRECTIONS

Our initial efforts in adapting RS have resulted in improvements in intervention acceptability and accessibility for the cultural group already included in current RS work (i.e., Latine families and health workers). With each study, we have progressively taken steps to increase the cultural congruence of the intervention for specific groups via increased levels of community input. The protocol used in the most recent iteration of the RS studies (*El Corazón de la Comunidad*) was developed in direct response to community input – and reflects the wisdom of the promotores and CHWs. Yet even with these modifications, there are several aspects of RS that could be targeted for future adaptations, as we discuss below. It is also worth mentioning that we have not evaluated whether the culturally adapted versions of the RS protocols are better received or more effective than the original version. We assume that because we adapted the intervention based on community feedback, the resultant product would be more acceptable and more effective, but we have not formally tested this assumption. Additionally, in considering cultural congruence, it has also become clear that it is important to consider generational or acculturative status, age, and other factors that may influence the degree to which Latine cultural values may resonate with clients.

Looking forward, from a theoretical perspective, we believe the three key areas related to RS interventions that could be more finely crafted to enhance intervention acceptability and cultural congruence are emotion regulation, savoring focus, and targets of RS. One important area that is relevant to RS is how emotions are regulated (i.e., experienced, recognized, and expressed) across cultural groups. Emotions are experienced differently across cultures (Immordino-Yang et al., 2016). For example, Western groups (Eid & Diener, 2001), African and African American samples (Kim-Prieto & Eid, 2004), as well as people of Latine heritage (Senft et al., 2021) have been found to emphasize positive emotional states while suppressing or avoiding negative ones. However, in individualistic cultures, suppressing negative emotion does not reduce the experiential or physiological effects of negative emotion (Gross, 2002). On the contrary, in East Asian collectivist societies, suppression of negative emotion is practiced in order to maintain the social order, but without negative effects on adjustment (Matsumoto et al., 2008). Still, other studies have shown that in East Asian societies, people emphasize harmony between positive and negative emotional experiences, valuing both as crucial parts of human perception (Sundararajan & Averill, 2007). The ways emotion is expressed in language can also heighten cultural differences (Goddard, 2002). Taking these differences into account, Smith and colleagues (2019) suggested that instead of seeking to amplify positive emotions, savoring interventions implemented with individuals from East Asian backgrounds may wish to emphasize dampening negative emotions. Alongside this argument, we wonder whether using terms that connote low arousal positive emotion (such as “calm” or “peaceful”) rather than terms that connote high arousal positive emotion (such as “excited” or “energizing”) would resonate more with people from East Asian backgrounds (Senft et al., 2021).

To address differences in emotional experience and expression, RS interveners could also use culturally congruent terms that better reflect how positive relational dynamics are encoded by individuals from certain cultures. For instance, Smith and colleagues (2019) noted that compared to other forms of savoring, RS may be particularly well-suited for people from East Asian backgrounds given its emphasis on social connectedness. Additionally, some linguistic modifications could enhance its appeal. For example, “honored” may be more congruent with filial piety values (Wong & Chau, 2006) than terms like “connected,”; changing terms to match cultural values will likely increase uptake of intervention. More generally, because impaired emotion regulation is a core process that has been implicated in various psychological disorders, and improvement in emotion regulation is a common focus and byproduct of many psychological therapies (Gratz et al., 2015), a comprehensive understanding of how emotion regulation processes unfold in different contexts for members of different cultural groups is essential to developing efficacious treatments.

Yet another aspect of RS that may be influenced by cultural experiences is the individual’s focus while savoring. Culture shapes one’s sense of self (e.g., degree of interdependence or independence) and how the self is experienced (Markus & Kitayama, 2014; Oyserman & Lee, 2008), which could influence the most appropriate focus of savoring. For selves that are interdependent with close others (e.g., family members), a relational form of savoring may be most appropriate and relevant (Smith et al., 2019). However, in cultural contexts that value interdependent value systems, family and community -- entities beyond the dyad -- are prioritized (Campos et al., 2007), suggesting that savoring experiences that focus on community-level interactions (e.g., such as the form of RS included in the *El Corazón de la Comunidad* study) would be appropriate. On the contrary, cultures that emphasize an independent sense of self may find self-focused savoring (i.e., personal savoring) to be more acceptable and beneficial. Indeed, several of our studies have documented the beneficial effects of personal savoring (e.g., Borelli, Kerr, et al., 2022; Pereira et al., 2021). Thus, future implementations of RS should take into consideration the kinds of relationships that are most salient or relevant to the target community.

Finally, diversifying the specific groups who receive RS (i.e., the targets of the intervention) will increase our knowledge of how modifications to RS achieve cultural congruence. To date, the majority of the studies conducted with RS have involved female participants (e.g., mothers or majority female samples). Female-identified people tend to feel more comfortable both giving and receiving care than male-identified participants, who may experience more difficulty in participating in this intervention due to differences in gender socialization (Chodorow, 1979; Gerson, 1985; Medved et al., 2006). It is noteworthy, however, that some relationship-based cultural values associated with Latine groups may also play a protective role. The culturally-rooted masculine norm of machismo consists of two components: *machismo* (i.e., the cultural values that emphasize attributes such as aggression, control, strength, and domination) and *caballerismo* (i.e., acting in a “gentlemanly” manner or displaying virtues like charity, honesty, honor, and politeness, especially toward women). This second component has been associated with affiliation and supportive co-parenting, processes likely linked to attachment-building and positivity in family relationships (Arciniega et al., 2008). Although we have not studied this in the context of RS, we wonder whether we could achieve greater cultural congruence with the values of Latine men by emphasizing a male role in protection, affiliation, and co-parenting (Arciniega et al., 2008). Despite this theorizing, our data on men’s response to the intervention are limited. Anecdotal evidence and pilot data from male participants who have completed RS interventions suggest that RS is acceptable for male-identifying participants (e.g., Wang, Bouche, et al., 2020; Wang, Huang, et al., 2020). Nevertheless, future research should investigate if modifications in language or approach would make RS more accessible and acceptable to a wider swath of the population.

It is important to consider that the process of making RS more adapted to Latine communities may have rendered the intervention less congruent with the values of other communities. Importantly, this assumption has not been tested. We are currently in the planning stages of projects that involve working with Black/African American families, Asian/Asian American families, as well as male clients. When beginning these projects, we are faced with the question of whether we start with the original version of RS (nonadapted version), with the version that was adapted for Latine families, or whether the projects should begin by seeking input on the intervention from the community through a process of co-development, as was done with the *Confía en Mi, Confío en Ti* project. In making these decisions, we consider the cultural values of the groups, their similarity with Latine cultural values, and the potential benefits and costs of each different approach.

## WHEN ADAPTING IS INSUFFICIENT

Although the goal of cultural adaptation is to modify protocols so that they are suitable for people with different backgrounds and needs, it is worth considering that RS may be a poor fit for certain clients. For instance, people who experience significant adverse life events may struggle with this approach. For individuals with past or current relationship challenges, reflecting on moments of positive connectedness that seem more inconsequential (e.g., a positive interaction with a medical provider) when they are dealing with ongoing interpersonal struggles may ignore the emotional reality of their experiences. Imagine clients who are involved in relationships with a high level of conflict, coercion, hostility, or abuse. RS may be contraindicated for this type of client – assisting them in focusing on moments of perceived safety when the relationship is unsafe would be a disservice to them. In these cases, assisting clients in developing accurate appraisals of the threat in the relationship and engaging in safety planning may be more helpful; that is, ensuring a client’s safety ought to be the primary treatment goal.

Further, RS may be difficult for clients who struggle to accept care – the double gaze of giving and receiving can pose a significant challenge for clients with this profile (e.g., Plys et al., 2020). In this circumstance, the intervener will need to assess whether RS leads the client in a direction that is beneficial for them, even if challenging. In other words, if being able to accept care is a treatment goal, increasing the client’s comfort with the thought of being cared for may be pursued through RS. However, the process of engaging with RS may need to be achieved more gradually, perhaps breaking down the steps more slowly, beginning by having the client savor moments of positive connectedness and gradually moving into savoring moments of being cared for in more and more vulnerable situations.

These considerations underscore a broader point – the need for the intervener to use clinical judgment about the appropriateness of the goals of RS for any individual client: to strengthen their mental representation of a relationship as loving, safe, and secure (care-receiving RS), or of the self as sensitive and responsive (care-giving RS). RS should be selected as an intervention when the therapeutic goal is to increase feelings of felt security within a given relational context, but first, the intervener must ask themselves whether that is a worthy goal. Interveners should assess for aspects of a client’s current or past experiences that could be relevant to their suitability to benefit from RS prior to administering the intervention. They can do so using self-report questionnaires (e.g., Traumatic Life Events Checklist-Revised; Kubany et al., 2000; Domestic Conflict Inventory; Margolin et al., 1998) or, in the case of an ongoing therapeutic relationship, their knowledge of the client. In these circumstances, interveners may wish to make one of several clinical decisions: (1) choose a different intervention approach, (2) administer RS but check in more frequently with the client regarding their reaction to the intervention, or (3) consider modifying RS in such a way as to preserve the intervention’s intention – helping the client connect with the experience of felt security, however that may be achieved. For instance, some clients may benefit more from examining relationship interactions from the past or hypothetical, imagined relationships (for an example of this type of modification, see the *Confía en Mi, Confío en Ti Study* description above).

## CONCLUSION

RS has shown promise not only in terms of its initial outcomes but also in terms of its accessibility and acceptability for members of Latine communities that have been marginalized and poorly served by health care systems. Other marginalized communities who are underserved by mental health interventions will be our next focus. Given its brief format and flexible structure, RS is also positioned for frictionless uptake by interveners and lends itself toward a culturally humble stance. Our hope is that our collaborative process of enhancing the cultural congruence of RS for members of the Latine community will inform similar efforts with other therapies that draw on the existing strengths of underserved populations. By listening to the communities we serve, we strengthen not only the collaborative process but also the interventions themselves.

## Figures and Tables

**TABLE 1:** Overview of the cultural and accessibility adaptations of the three studies.

Study	Cultural adaptation	Accessibility adaptation
PARENT Study	N/A	Using brief, at-home visits during evening hours. RS delivered by paraprofessionals (undergraduates, post-baccalaureate research assistants, developmental psychology masters’ students).
Confía En Mi, Confío En Ti	Co-developing the intervention reflecting the values of *familismo and simpatía*. Introducing RS with a script. Including a handout with images and a few words. Modifying RS intervention for individuals with trauma exposure. Changing to a group format. Delivered in Spanish. Including cultural metaphors used by the community. Delivered by *promotores (lay health workers)*.	Reducing the length of the intervention. Introducing RS with a script. Including a handout with images and few words. Changing to a group format. Translating RS and delivering RS in Spanish. Delivered by *promotores*.
El Corazón De La Comunidad	Revising the protocol based on interviews with CHWs (promotores and others who work at the community agency). Including themes of interest reflective of CHWs’ encounters with community members: secure base (e.g., supporting community members in achieving goals), safe haven (e.g., providing help to community members in time of need), moments of connectedness (e.g., feeling close to community). members).	Conducting virtual sessions. Delivered by paraprofessionals (undergraduates, post-baccalaureate research assistants).

## Footnotes

1 The supervision and training model for paraprofessionals is outside the scope of the current article, but includes weekly supervision from a licensed mental health professional. Cases that pose challenges receive additional resources (e.g., supplemental one-on-one sessions with a licensed mental health professional). These cases can include those related to the internal characteristics of a participant (e.g., individuals who have a significant trauma history or certain forms of psychopathology) or external characteristics (e.g., the individual is in a relationship with an abusive person, the individual is divorced or in the middle of a custody battle). In clinical settings with higher acuity, the use of paraprofessionals may not be clinically indicated (see Borelli, 2024 for more details); in such cases, delivery of RS by professionals may be not only recommended but the only ethical mode of administration. The RS treatment manual includes advice for assessing risk factors and adapting the protocol for higher risk clients.

2 We have conducted studies in which clients have savored their relationship with their young child, their partner, their parent or caregiver, their community, their sibling, or their friends.

3 Readers interested in the manual can email the first author. Borelli, 2024 also provides the manual and additional details on the administration of RS.

4 It is worth noting that in cases in which the client presents negative emotions that cannot be effectively contained by the intervener, this may indicate that the intervener should select a different intervention approach for the client. Likewise, in situations in which the client possesses certain internal or external risk characteristics, discussed in Footnote 1 above, which may not be revealed until this stage of the intervention, the intervener may decide this approach is not clinically indicated. At this point, the intervener can switch directions and instead focus on a topic or goal that is more accessible to the client. For instance, if the client is becoming dysregulated by the topic, the therapist may wish to transition to an approach that would help the client reduce or manage their arousal (e.g., teaching or administering a relaxation or mindfulness intervention). Alternatively, if the client finds it too challenging to disengage from negative emotional content (i.e., this content is too preoccupying), the therapist may wish to use techniques to help the client focus on and express these feelings (e.g., techniques from emotion-focused therapy). The therapist could then return to using an RS approach later if the client shows readiness to engage in the intervention. A lengthier discussion of this topic can be found in Borelli, 2024. We recommend that RS interveners conduct screening procedures to determine contraindications prior to delivering RS. While no standard protocol for screening exists at the current time, mindfulness meditation researchers have identified several potential safety issues related to mindfulness meditation practices in the research setting, including mental, physical, and spiritual health concerns (Lusty et al., 2009). Lusty and colleagues proposed screening schematics (e.g., for PTSD) and decision trees that may guide this process and could be useful in RS as well.

5 We use the term Latine in this article to refer to people who identify as Hispanic or Latine/o/a/x. We have chosen this term because it is the preferred term of our community partner. We are aware that people belonging to these groups identify by many different terms and that language, including language relating to group membership, has important implications.

6 Note that our initial work with Latino Health Access in the context of the Confía en Mí study was primarily conducted with promotores, one category of community health worker (CHW) – promotores are CHWs who engage in outreach. However, for the Corazón study, we broadened our work to include all CHWs because this was an area of need identified by our home community agency, Latino Health Access.
